# Effect of Reconstruction Algorithm on the Identification of 3D Printing Polymers Based on Hyperspectral CT Technology Combined with Artificial Neural Network

**DOI:** 10.3390/ma13081963

**Published:** 2020-04-22

**Authors:** Zheng Fang, Renbin Wang, Mengyi Wang, Shuo Zhong, Liquan Ding, Siyuan Chen

**Affiliations:** Instrument and Electrical Department, School of Aerospace Engineering, Xiamen University, Xiamen 361102, China; fangzheng@xmu.edu.cn (Z.F.); wangrenbin@stu.xmu.edu.cn (R.W.);

**Keywords:** X-ray absorption spectroscopy, hyperspectral CT, 3D printing polymer, material identification

## Abstract

Hyperspectral X-ray CT (HXCT) technology provides not only structural imaging but also the information of material components therein. The main purpose of this study is to investigate the effect of various reconstruction algorithms on reconstructed X-ray absorption spectra (XAS) of components shown in the CT image by means of HXCT. In this paper, taking 3D printing polymer as an example, seven kinds of commonly used polymers such as thermoplastic elastomer (TPE), carbon fiber reinforced polyamide (PA-CF), acrylonitrile butadiene styrene (ABS), polylactic acid (PLA), ultraviolet photosensitive resin (UV9400), polyethylene terephthalate glycol (PETG), and polyvinyl alcohol (PVA) were selected as samples for hyperspectral CT reconstruction experiments. Seven kinds of 3D printing polymer and two interfering samples were divided into a training set and test sets. First, structural images of specimens were reconstructed by Filtered Back-Projection (FBP), Algebra Reconstruction Technique (ART) and Maximum-Likelihood Expectation-Maximization (ML-EM). Secondly, reconstructed XAS were extracted from the pixels of region of interest (ROI) compartmentalized in the images. Thirdly, the results of principal component analysis (PCA) demonstrated that the first four principal components contain the main features of reconstructed XAS, so we adopted Artificial Neural Network (ANN) trained by the reconstructed XAS expressed by the first four principal components in the training set to identify that the XAS of corresponding polymers exist in both of test sets from the training set. The result of ANN displays that FBP has the best performance of classification, whose ten-fold cross-validation accuracy reached 99%. It suggests that hyperspectral CT reconstruction is a promising way of getting image features and material features at the same time, which can be used in medical imaging and nondestructive testing.

## 1. Introduction

As an additive manufacturing technology compared with conventional manufacturing processes which remove material from a workpiece, 3D printing based on melting and solidification has advantages of high precision, nanometer resolution, low material losses, low-level roughness, convenience of assemblage and so on [1,2]. Fused deposition modeling (FDM) and stereolithography (SLA) are two kinds of commonly used 3D printing techniques. FDM can enhance fracture toughness of parts by extrusion deposition trajectories improvement, and SLA is able to make thin wall structure with high-quality surface roughness [3]. 3D printing supplies as the material basis of this revolutionary prototyping technology, consequently, have received extensive attention. 3D printing’s material descriptions mainly contain polymer materials, metal materials, ceramic materials, and so forth. Of all the materials being applied to 3D printing, polymer materials have been widely used to medicine because of their high biodegradability and biocompatibility. For example, orthopedics applies biocompatible polymer scaffolds planted with tissue-specific cells to bone tissue engineering [4], biodegradable vascular stents wielding biodegradable polymer for the treatment of coronary artery disease can carry out degradation naturally over several months preventing complications resulted from long-term mucosal irritation or extraction [5], mixture of biodegradable polymers and hydrogels can be used to manufacture human-scale tissue for medicine by the integrated tissue-organ printer [6]. In medical imaging, polymer materials have acceptable X-ray transmission performance, no metal artifacts, and no impact on images leading to facilitate the detection of postoperative recovery. On the other side, polymer materials are infrequently applied to load-bearing parts because of their compliance, weakness, and anisotropic behavior. Therefore, in engineering, processor can ameliorate the mechanical properties of the polymer by adding fibers reinforcements, which is able to improve its strength and stiffness significantly [7]. Fiber-reinforced polymer can be broadly used for manufacturing industry such as civil construction, power electronics circuit boards, ships, wind turbine blades, unmanned aerial vehicles (UAVs), and automotive [8,9]. However, polymer filaments required for 3D printing demand optimum mechanical properties, flexibility, thermal stability, and proper melt viscosity being capable of passing through the printer nozzle [10]. The former study has proven that growth of carbon fiber (CF) content added into polymer can increase the viscosity of the material by three times resulting in reinforcing fibers clog, compromising the printability of materials [11]. Hence, proper amounts of additives are crucial for mechanical properties improvement of polymer structural materials, which have more industrial applications in contrast to conventional fiber-free polymers.

X-ray CT (XCT) has acquired incremental recognition for the inspection of internal structure of specimens. It is well-informed that XCT is a non-invasive radiological imaging technique since it has been widely used in medical imaging and industrial detection. XCT has the capabilities of detecting material structure with size of a few millimeters, dimensional measurements, and material characterization [12]. Thereinto, synchrotron X-ray source microtomography systems can attain the image resolutions in the micrometer range, thus improving the detection accuracy to examine microstructural cracks in the object [13,14]. Kowaluk et al. applied the developed method in relation to the ISO50% automated method to volume test workpieces improving the accuracy of linear dimension measurements and reducing relative measurement errors [15]. Davis et al. used X-ray microtomography systems to measure the linear attenuation coefficients of hydroxyapatite discs by scanning compared with the data that computed from their masses and volumes, discovering that the absolute accuracy between linear attenuation coefficients and the computed data was better than 1% [16]. These valuable applications determine that XCT is a promising imaging technique in structural detection. 

In the process of X-ray generation, the gradual deceleration of the high-velocity electrons produces a continuum, rather than X-ray of single wavelength corresponding to the tube voltage. Therefore, X-ray being presented in the form of spectrum consisted of two parts, one is a continuum with continuously varying wavelengths, and the other is a linear spectrum with discrete wavelengths. By applying X-rays with energy greater than the binding energy of the electrons of a certain element to the sample, X-ray photons ionize inner electrons into photoelectrons mainly through photoelectric effect and Compton scattering effect leading to characteristic X-ray with mutation of absorption coefficient, which is known as absorption edge. Characteristic radiation with absorption edges imply material identification information. Consequently, X-ray absorption spectroscopy (XAS) has become an effective material composition analysis method along with the essence of X-ray being uncovered. Particularly speaking, for Synchrotron XAS measurement, X-ray absorption fine structure (XAFS) analysis has been proven that it is an efficacious tool for studying the local electronic structure and atomic structure of materials [17,18,19]. Furthermore, recently study suggested that the details in the X-ray absorption near-edge structure (XANES) region accommodates information on multiple scatterers at a time, which can be used to identify the properties of the functional groups near the excited atoms and their relative positions [20].

With the growing requirement for non-invasive detection, it is gradually being explored that XCT has the potentiality of material characterization due to various energy of X-ray keeping different performance of attenuation characteristics. Compared with traditional XCT, X-ray spectral CT can obtain projections of objects under various energy channels in one scan, which makes it possible to distinguish the materials of the objects by extracting energy information. Fehrenbach et al. found that spectral CT providing quantitative values such as iodine content, spectral slope analysis, and attenuation increase can not only identify histological non-small cell lung cancer subtypes and signify their grading, but also have diagnostic value for lymph node metastasis assisting the differentiation of benign or malignant [21]. Schlomka et al. experimentally proved the feasibility of K-edge spectral CT by means of describing four attenuation processes (photoelectric absorption, Compton scattering, attenuation by iodine and attenuation by gadolinium) to carry out K-edge imaging of medical contrast agents [22]. Afterwards, Si-Mohamed Salim et al. further demonstrated that spectral CT has the capability of splitting two blended heavy metal-based contrast agents by qualitative and quantitative analysis in K-edge imaging [23]. Aamir Younis Raja et al. proposed the MARS MD algorithm quantifying material identification at multiple material concentrations by switching reconstructed energy bins into sparse material images [24]. As a result, spectral CT has already gained constant attention due to its capacity of tissue characterization, damage or lesion detection, and material decomposition.

Although multiple material decomposition algorithms have been proposed to improve contrast, spectral CT still has limitations on the component analysis of region of interest (ROI) on images. To make functional imaging practical, hyperspectral X-ray CT (HXCT) which is a method combining XCT with XAS makes full use of the ability of material characterization keeping in XAS and the capacity of structural imaging keeping in XCT [25]. However, the features of the reconstructed XAS by different reconstruction algorithms are distinct. It denotes that the quality of the reconstruction algorithm affects the characteristic form of the reconstructed XAS. Therefore, our study aims at the effect of various reconstruction algorithms on the classification results based on HXCT, to verify its feasibility. In this article, we employed hyperspectral CT to reconstruct images of training-set specimen including 7 kinds of commonly used 3D printing supplies and 2 different kinds of test-set specimens and then reconstructed the XAS of ROI in the CT images of all samples. One of the test-set specimens was the owner of 2 types of extra polymer besides the training-set specimen. Afterwards, we identified each polymer in test-set specimens from training-set specimen by means of classifying the reconstructed XAS of various polymers based on Artificial Neural Network (ANN).

## 2. Materials and Methods

### 2.1. Instrumental Setup and Specimen

**Figure 1:**
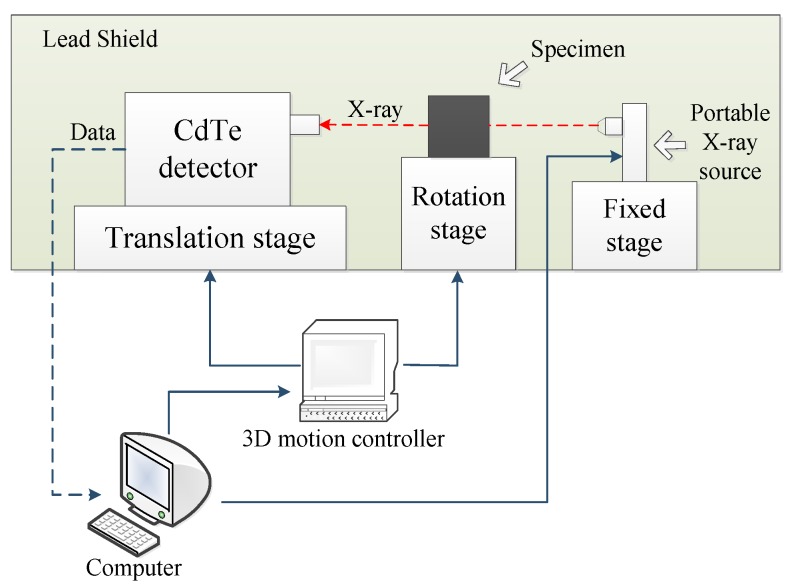
Hardware structure diagram of X-ray Absorption Spectrum acquisition system.

In this study, we adopted a XAS acquisition system possessing the capability of nondestructive spectral CT imaging and XAS detection developed by our laboratory as shown in Figure 1 [26]. This system mainly contains a portable X-ray source produced by Moxtek in the U.S.A, a CdTe photon-counting detector made by AmpTek in the United States, a rotation stage for specimen, a translation stage for the detector and other accessories. The source generates cone beam X-ray whose energy range from 4 keV to 60 keV with extreme power of 12 W and cone angle of 48°. The X-ray window is made of beryllium with thickness of 250 μm. The detectable area of CdTe detector is 9 mm^2^ with 100 μm thick beryllium window as well. The detector can record the spectrum of X-ray passing through the specimen on the rotation stage. The system can simulate linear array detector by continuous movement of single detector on the translation stage.

**Figure 2:**
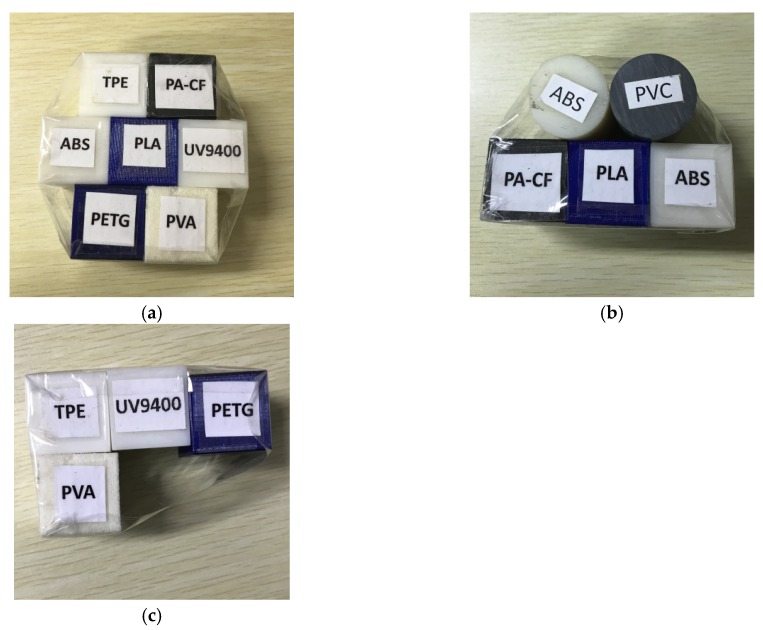
(**a**) Composition of training-set specimen including TPE, PA-CF, ABS, PLA, UV9400, PETG, and PVA (**b**) Composition of test-set specimen No.1 including PA-CF, ABS, PLA and two kinds of interfering samples ABS (cylinder), PVC (**c**) Composition of test-set specimen No.2 including TPE, UV9400, PETG, and PVA

7 kinds of 3D printing polymers applied in this study are shown in Table 1 which includes ingredients, abbreviations, density, and product’s information of all polymers. Each kind of sample was made into cuboid with length and width of 30 mm, height of 40 mm by FDM except UV9400 processed by SLA. First, we bundled up 7 kinds of 3D printing polymers listed in Table 1 by sellotape used to find out the suitable tube voltage and current for reconstructing a slice of specimen. Meanwhile, to prove the validity of the classification model, we randomly selected three kinds of polymers listed in Table 1, making one of them (ABS) a cylinder with a diameter of 30 mm to observe the effect of cross section on reconstructed XAS. Then we added an extra kind of polymer (PVC) with the same size of ABS cylinder into this specimen. The rest kinds of polymers listed in Table 1 was formed as another specimen. The composition of 3 kinds of specimens are shown in Figure 2.

### 2.2. Reconstruction of CT Image and XAS

**Figure 3:**
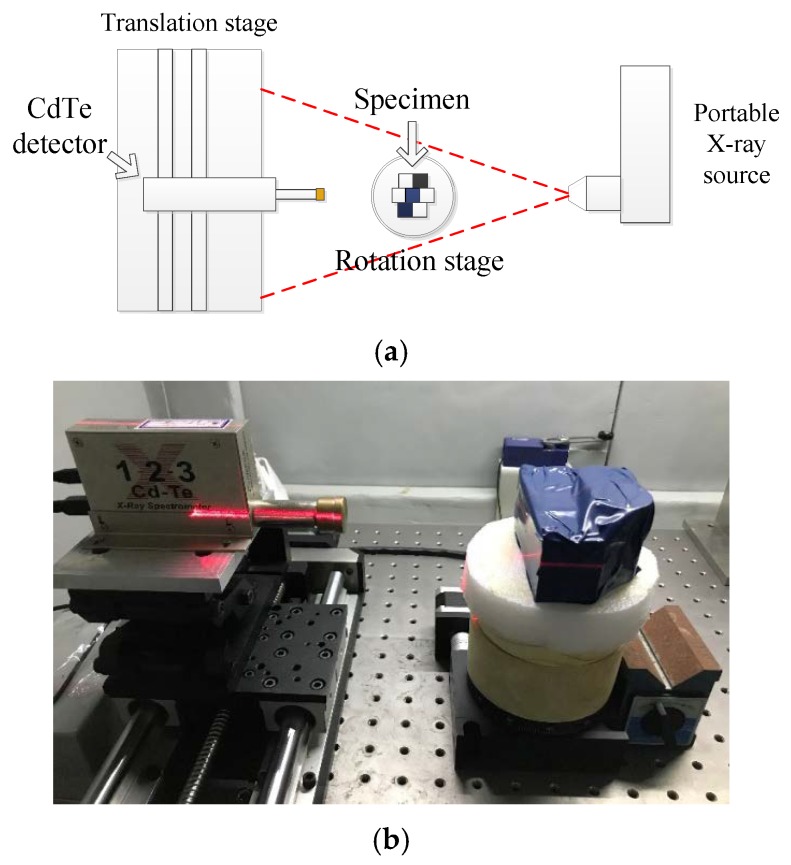
(**a**) Design of schematic of CT reconstruction experiment (**b**) Sealed specimen under experimental condition.

In this experiment, the instrumental position is demonstrated in Figure 3a. The distance between the source and the center of rotation stage was set to 450 mm. The distance between the detector and the source was set to 600 mm. The CdTe detector imitated a line array by moving within a range which was set to 120 mm. The full scan range was divided into 75 steps, which means the interval between adjacent steps was 1.6 mm. The detector moved a step after the rotation stage finished a full round divided into 72 steps, where the rotation stage rotated 5 degree per step. Every step the rotation stage being activated, the detector collected a XAS. The specimens were sealed with opaque tape as shown in Figure 3b.

In consideration of the fact that specimen (a) has the largest thickness, we chose it to pick out suitable tube voltage and current for preparation of CT reconstruction. Attenuation coefficient is a crucial parameter for CT reconstruction. When X-ray passes through an object with a thickness d, the X-ray undergoes absorption and scattering effect. Transmission intensity I and incident intensity $I_{0}$ can be attained from our experiment. Therefore, according to Lambert-Beer’s law [27], the attenuation coefficient μ is given by following formula:

(1) $$\mu =\frac{1}{d}\ln\frac{I_{0}}{I}$$

As the thickness of the specimen increases, the transmission intensity decreases gradually. To obtain a suitable attenuation coefficient value, it is critical to single out the tube voltage and current. Hence, we came up with a combination of voltage and current in Table 2, and collected XAS for comparison under the circumstance of the specimen with the maximum thickness. 

**Figure 4:**
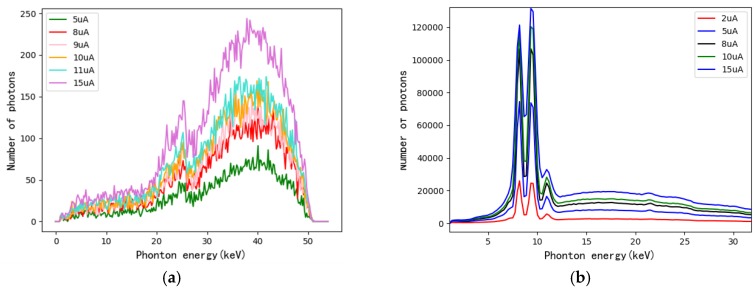
(**a**) Spectrum of specimen with maximum thickness (**b**) Spectrum of background.

Figure 4 shows the result of optimal spectral acquisition conditions test. It can be seen from Figure 4a that the noise of spectrum became larger and the number of photons decreased when we lowered the tube current. However, the spectrum of background as shown in Figure 4b revealed the information that it would blur the characteristic peak of spectrum when the tube current came to exceedingly large. Accordingly, to acquire a sufficient number of photons and to control the noise level, we chose 60 kV as the tube voltage and 11 μA as the tube current for obtaining XAS of CT reconstruction experiment.

As for reconstruction of images and XAS, Filtered Back-Projection (FBP) was employed in this experiment because it is the most widely used algorithm for commercial and medical CT applications [28]. In contrast to FBP on the quality of image reconstruction and the classification difference of reconstructed XAS, we also adopted ART and ML-EM as representative of iterative algorithm, which has been proven it is capable of reducing noise and obtaining agreeable imaging quality at low doses [29].

The basic idea of ART is to give an initial value of the reconstructed area, and then the residuals of projection are uniformly backprojected along the ray direction. Accordingly, the image is continuously corrected until the demand is met. ART can be expressed by the following formula [30]:

(2) $$x_{j}^{\left(k+1\right)}=x_{j}^{\left(k\right)}+\lambda^{\left(k\right)}\frac{a_{ij}\left(p_{i}-\sum_{m=1}^{M}a_{im}x_{m}\right)}{\sum_{m=1}^{M}a_{im}^{2}}$$

where $x_{j}^{\left(k+1\right)}$ is desired value of pixel, λ is the relaxation factor, k is the number of iterations, $a_{ij}$ is weighting factor of the jth pixel to the ith projection data. $p_{i}$ is ith projection vector.

ML-EM assumes that the photon beam emitted by the X-ray obeys the Poisson distribution, which reveals the emission pattern of spectral CT. The algorithm is operated by two step, calculating the conditional expectation of the log-likelihood function with respect to the measured projection data $p_{i}$ and the current pixel estimate $x_{j}^{\left(k\right)}$, then finding the maximum value of conditional expectation [31]:

(3) $$x_{j}^{\left(k+1\right)}=\frac{1}{\sum_{i}a_{ij}}\underset{i}{\sum }\frac{a_{ij}p_{i}}{\sum_{l}a_{il}x_{l}^{\left(k\right)}}x_{j}^{\left(k\right)}$$

where $x_{j}^{\left(k+1\right)}$ is jth pixel with estimation based on $x_{j}^{\left(k\right)}$, $\underset{l}{\sum }a_{il}x_{l}^{\left(k\right)}$ is the forward projection of pre-estimated data on the detector.

**Figure 5:**
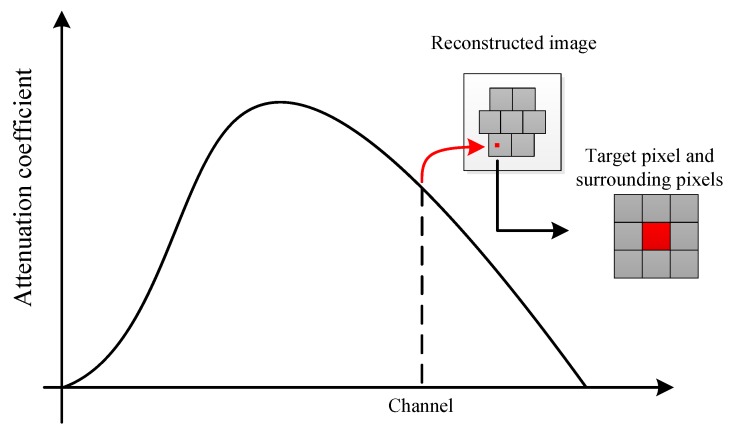
Theoretical approach of reconstructed XAS by Hyperspectral CT and definition of the target pixel and its surrounding pixels.

Spectral CT is a kind of polyenergetic image reconstruction, which denotes that X-ray is composed of multiple energy instead of single energy [32]. Moreover, hyperspectral CT can use point absorption coefficient of single-channel images to shape the reconstructed XAS, making an extension of the spectral dimension in spectral CT reconstruction [33]. The approach of reconstructed XAS is shown in Figure 5. To minimize the effects of reconstruction noise, every reconstructed XAS was formed by averaging the XAS of target pixel and its surrounding pixels.

### 2.3. Classification of Reconstructed XAS

Before the classification, the data preprocessing is quite important, which contains two steps. First, 0–1 normalization was applied for diminishing the error resulting from experimental factors as well as varying thickness of specimen due to rotation. 0–1 normalization [34] is given by:

(4) $$\mu^{\prime }=\frac{\mu -min}{max-min}$$

Secondly, dimensionality reduction reserves the most contributing features of high-dimensional data, removing noise and inconsequential features, thereby achieving the goal of improving data processing speed. Principal component analysis (PCA) [35] is a widely used method of dimensionality reduction of high-dimensional data while minimizing information loss [36]. Suppose the data set is matrix $X=\left[x_{1},x_{2},x_{3},\cdots ,x_{n}\right]$, then the process of centralizing the data set matrix would be:

(5) $$y_{i}=x_{i}-\frac{1}{m}\overset{m}{\underset{i=1}{\sum }}x_{i}$$

after centralization of data set, we can proceed the eigenvalue decomposition of covariance matrices $YY^{T}$, calculating the eigenvector $w_{i}$ corresponding the large contributing eigenvalue $\lambda_{i}\left(i\leq Q\leq n\right)$ to build projection matrix:

(6) $$W=\left[w_{1},w_{2},w_{3},\cdots w_{q}\right]\left(q\leq n\right)$$

where q is the number of the large contributing eigenvalue, which can be set by selecting a threshold t, and then we can obtain the minimum q  value that makes the following formula hold:

(7) $$\frac{\sum_{i=1}^{q}\lambda_{i}}{\sum_{i=1}^{Q}\lambda_{i}}\geq t$$

**Figure 6:**
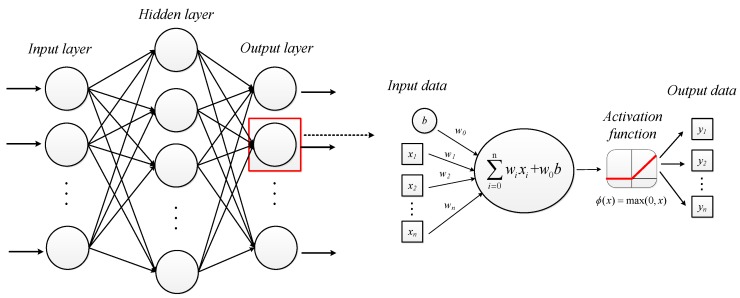
Structure and the principle of ANN, its details of neurons including activation function.

ANN is used to classify the reconstructed XAS after dimensionality reduction by PCA. ANN is also known as Multi-Layer Perceptron (MLP) composed of input layer, hidden layer, and output layer. Figure 6 demonstrated the process of ANN. The neurons in each layer receive input data that are passed through a weighted connection. The total input value received by the neuron would be compared with the threshold and then processed by activation function to generate the output of the neuron [37,38].

The threshold can be replaced by bias input b, $w_{i}$ is the connection weight [39], therefore the mathematical model of ANN is given by:

(8) $$y_{i}=f\left(\underset{i}{\sum }w_{i}x_{i}+w_{0}b\right)$$

The activation function of hidden layer we chose to use in this paper is Rectified Linear Unit (ReLU) [40] as shown in the following formula:

(9) f(x)=max(0,x)

ReLU makes the output of some neurons turning into zero, which causes the sparseness of the network, thereby reduces the interdependence of parameters contributing to alleviate the occurrence of over-fitting problems [41]. 

For model evaluation, k-fold cross-validation (kCV) is an option for our assessment. Due to the rationality of dividing the data set into the training set and test set, kCV makes the most of scant data by means of splitting the data multiple times for data reuse. Moreover, kCV can estimate the performance of the model relatively accurately, and select the best model by evaluating the generalization error [42]. To be more specific, kCV is used for model tuning, to help us find the parameters that optimize the generalization performance of the model. Once the model parameters are determined, they can be used to retrain the model on the training sets, then applied the model to the test set for making a final evaluation of the model performance. In addition, confusion matrix, sensitivity, precision, specificity, and accuracy as shown in Table 3 and the following formula, were also selected into model evaluation criteria:

Sensitivity, precision, accuracy and specificity [43,44] shown as following formulas can be used as an evaluation index of ANN’s performance in the case of multiple training and testing, or training and testing on multiple training sets.

(10) $$Sensitivity=\frac{TP}{TP+FN}$$

(11) $$Precision=\frac{TP}{TP+FP}$$

(12) $$Specificity=\frac{TN}{TN+FP}$$

(13) $$Accuracy=\frac{TP+TN}{TP+FP+TN+FN}$$

It should be point out that the division of the training set and test set was different from the previous. Given that all of the reconstructed XAS of Figure 2a was set to the training set, we reconstructed the CT images and XAS of Figure 2b,c used for the test set, which means the training and test set are no longer limited to an identical CT image.

## 3. Results and Discussion

### 3.1. Reconstruction of CT Image and XAS in ROI

**Figure 7:**
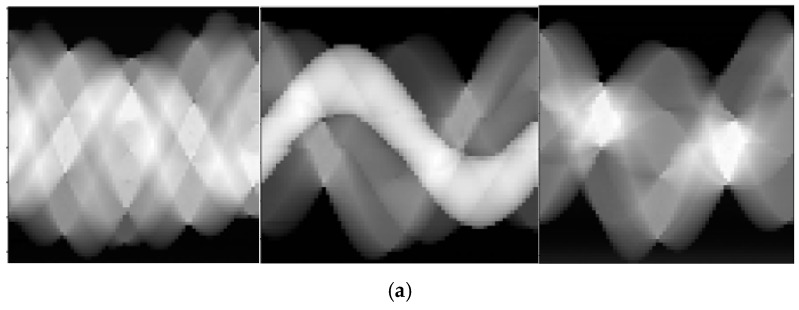

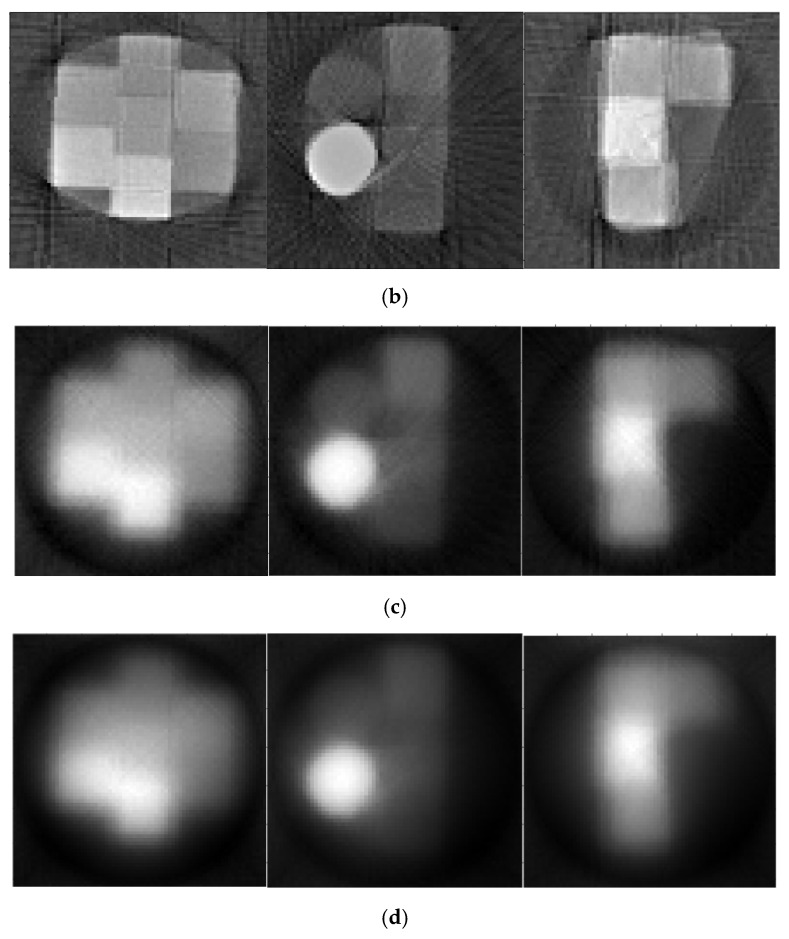
(**a**) Sinograms of all specimens by FBP and reconstructed CT images of all specimens by FBP (**b**), ART (**c**) and ML-EM (**d**).

Figure 7 shows the result of CT images reconstructed by FBP, ART, and ML-EM. In the perspective of reconstruction algorithm, FBP has the best structural imaging results, where the edges of each sample are clearly visible but artifacts caused by the instability of photon-counting detector during the data acquisition process and beam hardening effect are also getting apparently on the images (Figure 7b). In contrast to FBP, ML-EM eliminates streak artifacts on the images while it compromises the structural features. The edges among each sample in ML-EM figure become blurry (Figure 7d). The consequent of ART falls in between yet it has other defects that the reconstructed image has linear artifacts since ART is a line-by-line iteration method [45], theoretically (Figure 7c).

**Figure 8:**
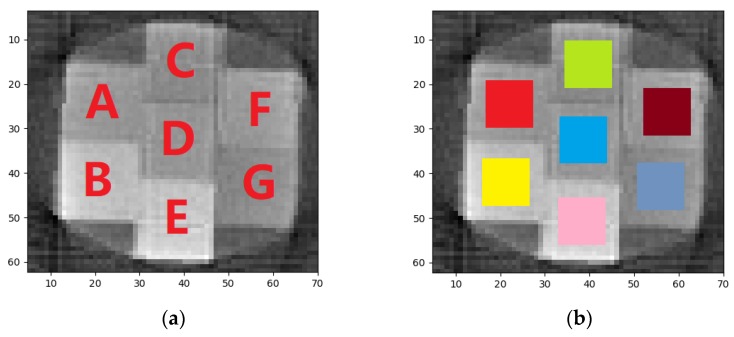
(**a**) Markers of each kind of samples in training-set specimen (**b**) ROI distribution of CT image by FBP.

**Figure 9:**
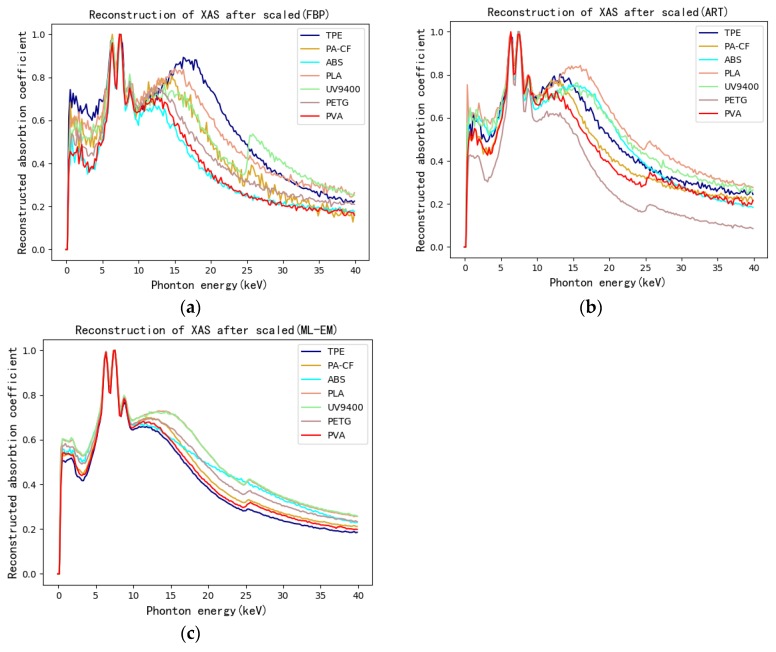
(**a**) Reconstructed XAS of ROI on the CT image of training-set specimen by FBP and ART (**b**) as well as ML-EM (**c**).

**Figure 10:**
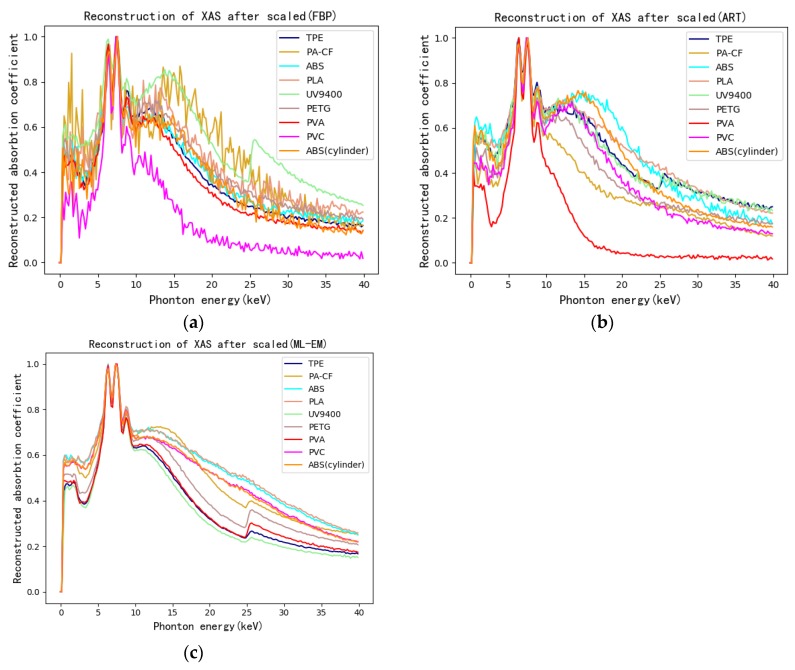
(**a**) Reconstructed XAS of ROI on the CT image of test-set specimens by FBP and ART (**b**) as well as ML-EM (**c**).

Taking the figure of FBP as an example, the ROI on the CT image of training-set specimen is shown in Figure 8. The specific pixel coordinates are shown in Table 4. The XAS of pixels in ROI can be obtained by hyperspectral CT method. Every piece of ROI represents corresponding 3D printing polymers in specimen. Five pixels were selected as central pixels in each ROI. The reconstructed XAS of each central pixel and its surrounding eight pixels were then obtained. To make the amount of training-set XAS appreciable, four reconstructed XAS of eight surrounding pixels were picked out in addition to the reconstructed spectra of the central pixel. As a result, five reconstructed XAS were averaged to obtain a XAS considered to be the reconstructed XAS of central pixel. In this way, each ROI can gain 350 reconstructed XAS. The results of reconstructed XAS after normalization are shown in Figure 9. The reconstructed XAS of the test-set specimens can be obtained by the same method as shown in Figure 10.

Considering the performance of various algorithms from the perspective of reconstructed XAS as shown in Figure 9 and Figure 10, FBP has stronger noise, yet retains the most spectral features at the same time compared to iterative algorithm. However, between iterative algorithms, ART keeps more features and has stronger noise while ML-EM lowers the noise of images yet blurs the structure. It is because iterative methods can improve image quality and reduce image noise compared with FBP being a kind of analytical methods [46]. Therefore, the quality of the reconstructed XAS is related to the image quality. On one hand, the image structure grows to be clearer, the reconstructed XAS keeps more spectral features. On the other hand, there is positive correlation between image noise and reconstructed XAS noise. 

### 3.2. Reconstructed XAS Preprocessing and ANN

**Figure 11:**
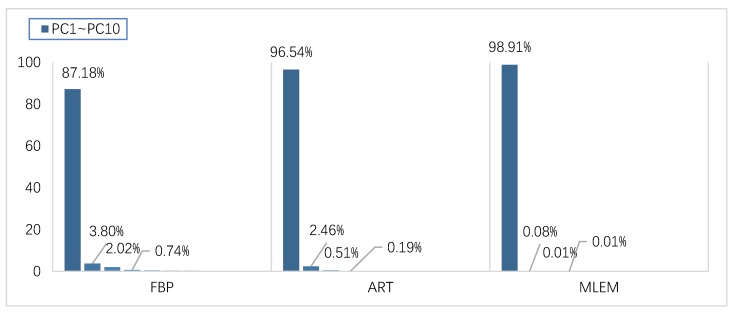
The first 10 principal component contribution rates of three kinds of reconstruction algorithms.

**Figure 12:**
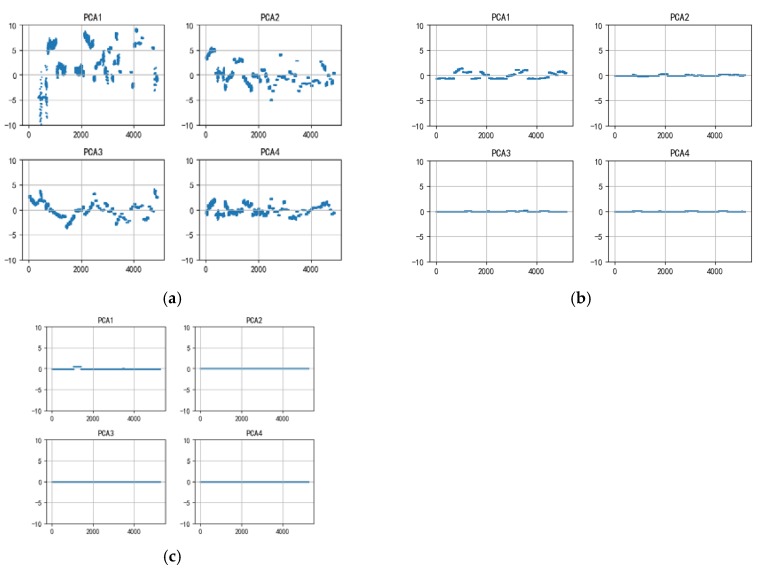
(**a**) The first 4 principal component distributions of the reconstructed XAS under FBP and ART (**b**) as well as ML-EM (**c**).

By means of hyperspectral reconstruction, the ROI of the train-set specimen was spectrally reconstructed. Each specimen obtained 350 reconstructed XAS, which means every kind of reconstruction algorithm possessed the same number of the training-set size including 2450 spectrum. The test-set spectrum was obtained from the ROIs of the test-set specimen NO.1 and NO.2. After the outliers were eliminated, the test-set size of FBP, ART, and ML-EM was respectively 2450, 2730, and 2800. PCA was used to perform feature extraction calculations on all spectrum including the test set and training set after 0–1 normalization. The contribution ratio distribution of the first ten principal components is demonstrated in Figure 11. It can be seen that PC1 contribution of the three algorithms have reached 87.18%, 96.54% and 98.91%, while the cumulative contribution of the top ten principal components of FBP, ART, and ML-EM have reached respectively 95.94%, 99.83% and 99.98%, explaining almost everything in reconstructed XAS. The distribution of the first four principal components is shown in Figure 12.

As can be seen from Figure 12, the principal components distribution of the same polymers has a certain variance in the FBP and ART, yet the variance becomes smaller in ML-EM. It is further proved that the XAS reconstructed by FBP and ART has certain noise interference, and the anti-noise effect of ML-EM is better than the previous two algorithms, but the variance of the main component distribution of XAS reconstructed by ML-EM turn to small among different polymers in every specimen, which is going to compromise the results of classification. Based on the contribution rate of principal components, the first four principal components of reconstructed XAS were selected to apply to the next identification calculation. Therefore, ANN was trained by the first 4 principal components of the reconstructed XAS in the training set, then the trained ANN was used for identifying the reconstructed XAS in all test sets. The number of hidden layers and the number of neurons in the hidden layer were determined by the best results, evaluating by ten-fold cross-validation accuracy, from multiple times of training, which is illustrated in Table 5. It can roughly be seen that FBP gets the best performance of all reconstruction algorithm. The number of model layers and nodes become larger, the more complex the model is going to be, causing ANN being easy to overfit. With the reconstructed XAS by ART having inevitable streak artifacts, accordingly, the model learned the incorrect information in the data, which leads to have relatively bad outcome of the average accuracy. Therefore, we chose the double-layer ANN with 128 neurons per layer as training model.

### 3.3. Evaluation of Classification Results Based on ANN

**Figure 13:**
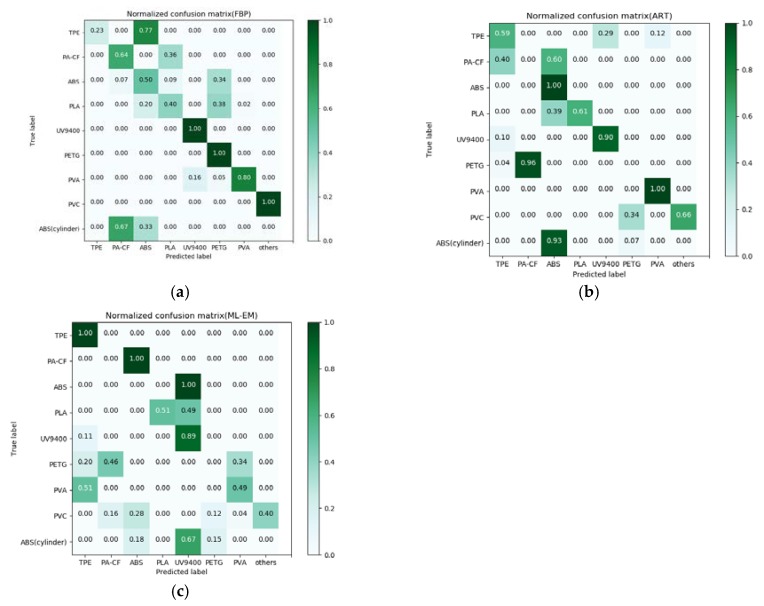
(**a**) Normalized confusion matrix of ANN under FBP and ART (**b**) as well as ML-EM (**c**).

The normalized confusion matrix results related to the test sets of three kinds of reconstruction algorithms through ANN trained models are shown in Figure 13. It can be intuitively seen from the figures that FBP has the best classification performance, except that the sensitivity of the three polymers, TPE, PLA, and ABS (cylinder), is less than 50%, and the rest perform quite well, of which the sensitivity of UV9400, PETG, PVA and PVC can reach more than 80%. The classification performance of the iterative algorithm is generally inferior compared with FBP. In the ART algorithm, PA-CF and PETG was incorrectly classified into other labels. Only ABS, ABS (cylinder), UV9400, and PVA have excellent classification performance, all reaching 90%. PVC as an interference sample excluded from the training set has 34% were misidentified as PETG. The performance of ML-EM is the worst. Only the sensitivity of TPE and UV9400 reach more than 80%, yet 60% of the reconstructed XAS of PVC was misidentified. It is worth mentioning that although ABS, ABS (cylinder) were wrongfully classified to other labels, more than 60% of the reconstructed XAS of them were classified in the same label. It means the model considered them as the same polymer, yet they are not classified to the correct label.

**Figure 14:**
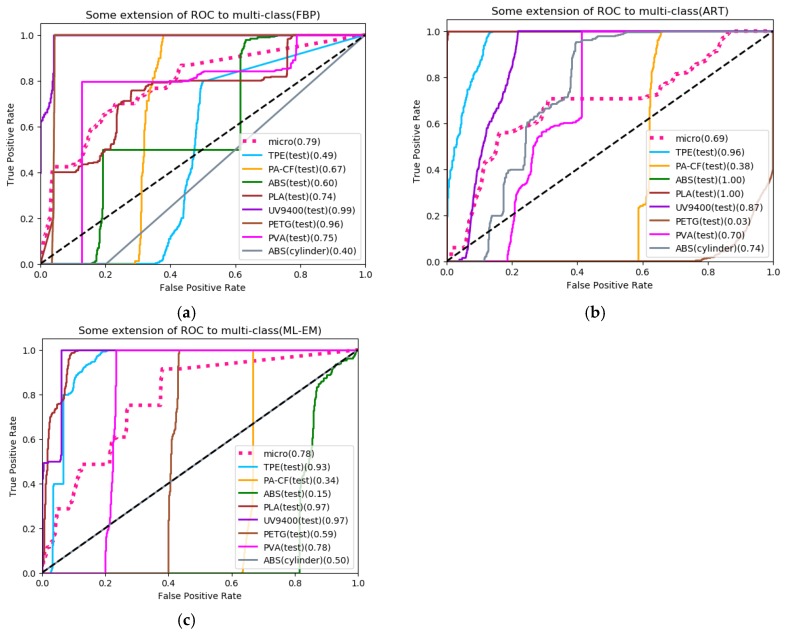
(**a**) ROC curves for prediction result of XAS reconstructed by FBP and ART (**b**) as well as ML-EM (**c**).

The model evaluation indicators which are sensitivity, specificity, precision, and accuracy are given in Table 6. It can be observed that the accuracy rate of the three reconstruction algorithms reach more than 70%; however, FBP still has the best evaluation performance. As for iterative algorithm, ART performs better than ML-EM. In addition, UV9400, PVA and PVC have over 80% sensitivity and precision at the same time in FBP’s evaluation, while ART only has PVA achieving the same, and ML-EM has none. It further illustrates that the model is more effective for FBP. The receiver operating characteristic (ROC) curves of the three reconstruction algorithms are shown in Figure 14. The value in parentheses after the label represents the area under curve (AUC). It can be seen that the average ROC curve of FBP has a maximum AUC value of 0.79, but the classification of ABS (cylinder) is not acceptable.

## 4. Conclusions

Given the performance of the reconstructed XAS classification results of three different reconstruction algorithms, it suggested that FBP was the algorithm with the best performance. Each polymer was identified varied in amounts but all of them were classified into the correct label, and the AUC value of the average ROC curve reached 0.79. In the case of iterative algorithms with low-resolution sampling, reducing the number of iterations when it must also take into account the efficiency of XAS reconstruction, it compromised the accuracy of image reconstruction and accordingly affects the accuracy of the reconstructed XAS. Although the classification results of three reconstruction algorithms was diverse, no matter which kind of algorithm was used, for ABS and ABS (cylinder), most of the reconstructed XAS of both were consistently classified into the identical label. It indicates that the reconstructed XAS of the same substance is considered to be the same, which proves that hyperspectral CT reconstruction immunes to the transformation of sample shape and thickness. The favorable result of another interfering polymer PVC denotes that the misclassification rate of distinguished out-of-database XAS is relatively low. In summary, results of this experiment prove that hyperspectral CT technique has the potential to detect the internal structure of the object and identify the components in it at the same time. Compared with the past experiments in which the reconstructed XAS of the test set and the training set were extracted from the same CT image, this experiment relieved the limitation between the training set and the test set. Further study will focus on improving the stability of the equipment, optimizing algorithms and noise control, and minimizing the effect of too many abnormal values of the reconstructed XAS caused by hardware conditions, artifacts, and noise as much as possible, making it possible to quantify the training set reconstructed by hyperspectral CT method into a reconstructed XAS library since reconstruction is no longer limited to identical figures. The research brings up a new idea for non-destructive testing and medical image pathological diagnosis.

## Figures and Tables

**Table 1:** Ingredients, abbreviations, density, and other information of 3D printing polymer.

Ingredients of Sample	Abbreviations	Density (g/cm^3^)	Trade Name	Manufacturer
99% ABS resin 0.5% N,N’-Ethylene distearylamide 0.5% Tris-(2,4-di-tert-butylphenyl) phosphite	ABS	1.080	ABS filament	eSUN
1,4-Benzenedicarboxylic acid, polymer with 1,4-cyclohexanedimethanol and 1,2-ethanediol	PETG	1.380	PETG filament	eSUN
55% Polylactide resin 35% Poly (DL- lactide) 5% DL-Lactide 5% L-Lactide	PLA	1.430	PLA filament	eSUN
Polyvinyl alcohol	PVA	1.310	PVA filament	eSUN
Thermoplastic elastomer	TPE	0.98	TPE filament	eSUN
UV Photosensitive Resin	UV9400	1.13	C-UV9400	Online personal supplier
74% Nylon-66 25% Carbon Fiber 1% Additives	PA-CF	1.24	ePA-CF filament	eSUN

**Table 2:** Test tube voltage and current of spectral acquisition conditions.

Tube Voltage (kV)	Tube Current (μA)					
60	2	5	8	10	11	15

**Table 3:** Definition of Confusion Matrix.

Predict	Real	
	True	False
Positive	True Positive (TP)	False Positive (FP)
Negative	False Negative (FN)	True Negative (TN)

**Table 4:** Coordinate descriptions of ROI in Figure 7 for each kind of polymers.

Mark	A	B	C	D	E	F	G
Material	TPE	PA-CF	ABS	PLA	UV9400	PETG	PVA
Row(X)	16–26	15–25	34–44	33–43	32–42	51–61	50–60
Column(Y)	18–28	36–46	10–20	27–37	45–55	19–29	37–47

**Table 5:** Elementary evaluation results affected by multiple parameters of hidden layer.

Reconstruction Algorithms	Parameters of Hidden Layer	Average Accuracy of kCV	Confidence Interval
FBP	(256)	0.99	(±0.02)
	(128 × 64)	0.99	(±0.03)
	(128 × 128)	0.99	(±0.02)
	(256 × 128 × 64)	0.99	(±0.02)
	(256 × 128 × 128)	0.98	(±0.02)
ART	(256)	0.67	(±0.17)
	(128 × 64)	0.68	(±0.20)
	(128 × 128)	0.63	(±0.20)
	(256 × 128 × 64)	0.48	(±0.26)
	(256 × 128 × 128)	0.58	(±0.25)
ML-EM	(256)	0.42	(±0.24)
	(128 × 64)	0.38	(±0.18)
	(128 × 128)	0.44	(±0.19)
	(256 × 128 × 64)	0.43	(±0.19)
	(256 × 128 × 128)	0.43	(±0.18)

**Table 6:** Evaluation parameters of prediction result by ANN.

Algorithm	Evaluation	TPE	PA-CF	ABS	PLA	UV9400	PETG	PVA	PVC	ABS (Cylinder)	Average
FBP	Sensitivity	0.23	0.64	0.50	0.40	1.00	1.00	0.80	1.00	0.33	0.66
	Specificity	0.89	0.93	0.77	0.97	0.96	0.90	0.99	1.00	0.78	0.91
	Precision	1.00	0.22	0.26	0.74	0.83	0.13	1.00	1.00	0.13	0.59
	Accuracy	0.89	0.92	0.76	0.89	0.97	0.86	0.96	1.00	0.75	0.89
ART	Sensitivity	0.59	0.00	1.00	0.61	0.90	0.00	1.00	0.66	0.93	0.63
	Specificity	0.93	0.86	0.74	1.00	0.96	0.94	0.98	1.00	0.80	0.91
	Precision	0.55	1.00	0.17	1.00	0.56	0.00	0.89	1.00	0.40	0.62
	Accuracy	0.89	0.74	0.75	0.95	0.96	0.82	0.98	0.96	0.81	0.87
ML-EM	Sensitivity	1.00	0.00	0.00	0.51	0.89	0.00	0.49	0.40	0.18	0.39
	Specificity	0.90	0.91	0.81	1.00	0.76	0.97	0.95	1.00	0.83	0.90
	Precision	0.57	0.00	0.00	1.00	0.16	0.00	0.56	1.00	0.08	0.37
	Accuracy	0.91	0.80	0.70	0.94	0.76	0.85	0.89	0.93	0.79	0.84
